# Human error risk prioritization in crane operations based on CPT and ICWGT

**DOI:** 10.1371/journal.pone.0297120

**Published:** 2024-02-01

**Authors:** Aihua Li

**Affiliations:** College of Mechanical Engineering, Yancheng Institute of Technology, Yancheng, 224051, Jiangsu, China; National University of Sciences and Technology, PAKISTAN

## Abstract

Human error plays a significant role in crane safety. To increase the accuracy and rationality of human error risk prioritization for crane operations, this study proposes a risk prioritization model for human errors in crane operations based on the cumulative prospect theory (CPT) and the improved combination weighting model of game theory (ICWGT). The ICWGT integrates the risk-factor weights obtained via subjective and objective methods. Trapezoidal fuzzy numbers are used to describe experts’ uncertainty information. Then, the CPT is applied to handle the assessment of experts’ risk attitudes in the decision process. The human error risk ranking of crane operations is obtained according to the overall prospect values calculated using the CPT. A case study of human error in overhead crane operations was conducted, and sensitivity and comparison analyses confirmed the feasibility of the proposed model. The proposed ranking mechanism for human error risk priority in crane operations is helpful for crane risk management.

## Introduction

**Fig 1:**
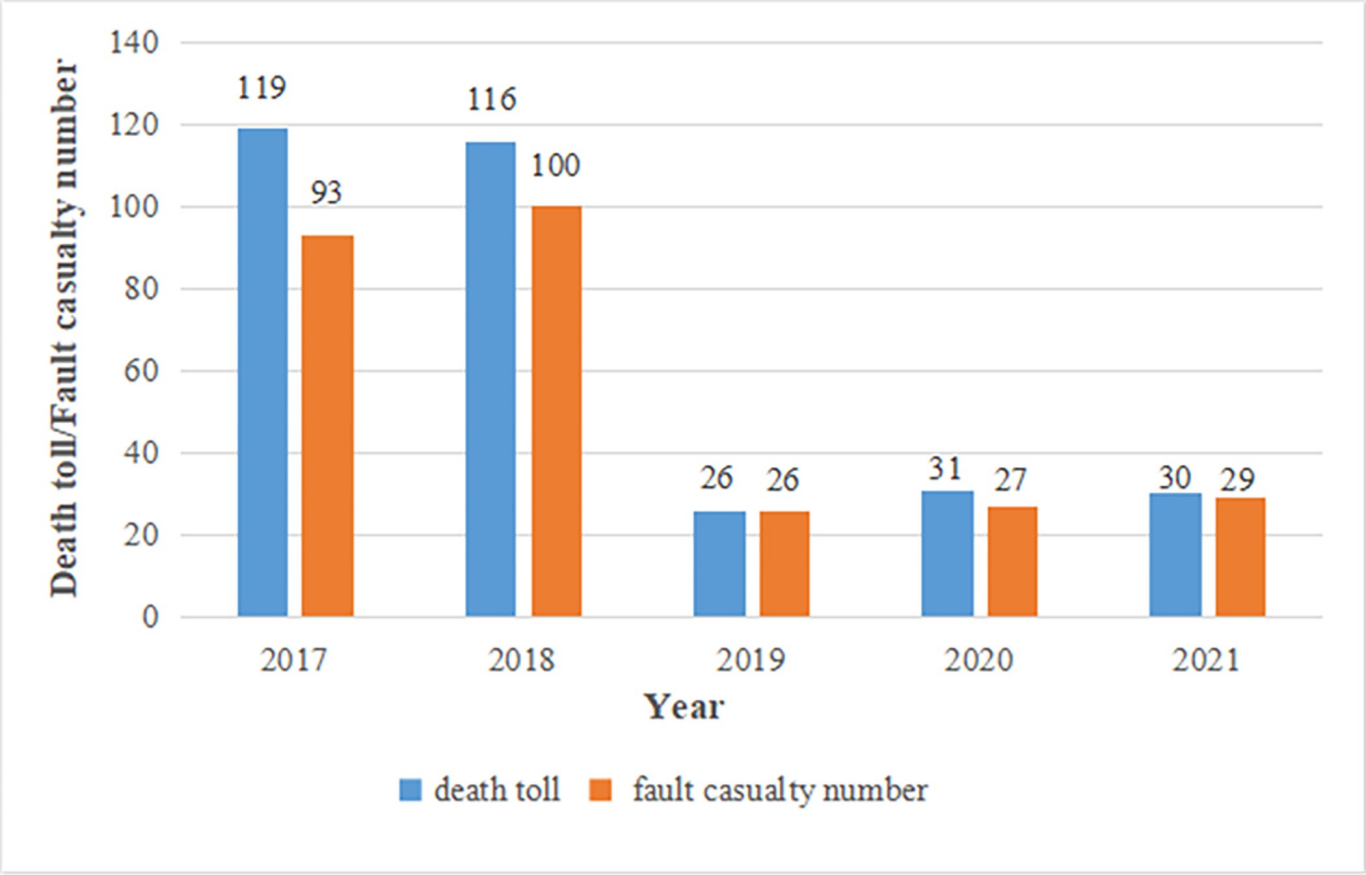
Crane death toll and fault casualty numbers in China (2017–2021).

Cranes are widely used in construction, dockyards, railway transportation, and other production fields for heavy load hoisting and conveying [1]. In 2021, 2.73 million cranes were registered in China [2]. Crane accidents account for a large proportion of accidents involving special equipment [3,4]. In 2021, 110 special-equipment accidents were reported in China, killing 99 people, of which crane accidents accounted for 26.36% [2] and were responsible for 30.3% of the total deaths [2]. As shown in Fig 1, crane accidents and death tolls exhibit a descending trend. However, crane safety remains an important issue in China. Between 2017 and 2021, 322 workers died in 275 crane-related accidents in China, with an average of 65 deaths per year. The average death toll was 51% higher than that in the United States from 1992 to 2006 [5]. Thus, the analysis of crane safety and risk is vital for preventing accidents and reducing casualties.

Cranes encounter several potential risk factors during the manufacturing process, such as structural deformation [6], human error [7], and fracture [8]. These risk factors may result in casualties, severe economic losses, and other adverse social effects. The main cause of crane accidents is improper and illegal human operations [2]. Thus, human error during operations plays an important role in crane safety analyses [7]. Analysis of failure risk is a crucial part of studying process safety to identify key failure paths, investigate potential risk factors, and ensure safe operations. Therefore, risk analysis of human error in crane operations is crucial for safe production.

Failure mode and effects analysis (FMEA) has been used in various fields [9]. It can prioritize potential failure modes for safety management and assign a risk priority number (RPN) as an index for evaluating the risk level of each failure mode. The RPN is determined by multiplying three factors: occurrence (O), severity (S), and detection (D) [10]. For example, to address the complex problems of a quayside container crane system, Zhang et al. [11] proposed a health monitoring method based on FMEA. Sun et al. [12] applied the fuzzy FMEA and ALARP (as low as a reasonably practical rule) methods to rank the potential risks of ship-to-shore container crane heightening. Although traditional FMEA methods have the advantages of simplicity and clarity, their limitations have attracted increasing attention. The most significant results are summarized below [13]. (1) The rationality of the RPN calculation is debatable. (2) The same RPN can be achieved using different combinations of O, S, and D, which is inconvenient for risk management. (3) It may be difficult to determine the precise values of O, S, and D. (4) The relative importance of these three risk factors is generally neglected.

In response to these deficiencies, many multi-criteria decision-making (MCDM) methods have been used for the risk prioritization of the failure modes of cranes. For instance, Li et al. [14] employed grey relational analysis (GRA) and fuzzy confidence theory to identify the risk of overhead cranes for metallurgical plants but did not consider the weights of O, S, and D. Raviv et al. studied dangerous situations and potential safety risks related to crane work and implemented an analytical hierarchical process (AHP) to evaluate the severity value of quantitative results, thereby calculating the total potential risk of each event. On the basis of an integrated MCDM approach combining hierarchical task analysis (HTA), the systematic human error reduction and prediction approach (SHERPA), and the VIseKriterijumska Optimizacija I Kompromisno Resenje (VIKOR) method, Mandal et al. [7] proposed a ranking method for quantifying the failure modes of human error risk in overhead crane operations. However, the risk-factor weights depend only on the subjective experience of experts, and the objective weights are ignored. Das et al. [15] created a scientific model that employed the Z-number and VIKOR methods, along with the concept of fuzzy AHP and the Shannon entropy principle, for the hazard prioritization of electric overhead traveling crane operations. To address the issue of risk management associated with the increased height of quay cranes, Zhao [16] proposed an improved FMEA approach for heightening quay cranes using entropy and GRA. By utilizing the FMEA of the construction process for heightening quay cranes as a case study to verify the effectiveness of the developed model, this study demonstrated that the method assists in reducing the impact of subjective uncertainty on risk classification and increases the precision of risk prioritization. In addition to the aforementioned MCDM technology for crane risk assessment, numerous effective and innovative methods have been widely implemented in various fields, such as complex proportional assessment (COPRAS) [17,18], the decision-making trial and evaluation laboratory (DEMATEL) technique [19], the technique for order of preference by similarity to ideal solution (TOPSIS) [20], evaluation based on distance from average solution (EDAS) [21,22], and data envelopment analysis (DEA) [23]. However, research on the use of these methods for crane risk assessment is limited.

In crane risk prioritization, the subjective weights and objective weights are rarely combined. Although scholars have noted this detail and adopted the combination weighting method to determine the integrated weights of risk factors, it is challenging to rationally distribute the proportions of various weights [24]. Therefore, it is necessary to develop a reasonable combination weighting approach for crane risk analysis. Methods for combination weighting have been proposed, e.g., maximizing the deviation model, maximizing the difference, the relative entropy method, the interval estimation method, mathematical programming, and the combination weighting model of game theory (CWGT). The CWGT is one of the most popular theories among these combination-weighting models and has been widely applied. However, there may be negative values of the weight coefficient after the calculation. This does not satisfy the assumption that all weight coefficients are positive. Moreover, a negative weight coefficient results in a negative weight value. This is unreasonable, because the index weight value must be >0. Fortunately, the improved combination weighting model of game theory (ICWGT) [25] proposed by Li can overcome these drawbacks. It has been applied to crane safety assessments [25] and effectiveness evaluations of electromagnetic missile launches [24]. Therefore, the ICWGT was applied to determine the combination weights of O, S, and D in this study.

Although the aforementioned efforts have contributed to addressing the disadvantages of human error risk prioritization for crane operations, experts’ risk attitudes are not involved in the entire risk analysis process. For addressing this issue, the MCDM method combined with prospect theory [26] has the ability to rank risk priority by reflecting the psychological behavior of experts. However, there is a critical defect in prospect theory, i.e., dominance violations. The cumulative prospect theory (CPT) [27] was developed to solve this problem and can be used for risk prioritization [28]. In this study, we applied the CPT to the risk analysis of human error in crane operations.

Herein, we propose a risk prioritization model for human error in crane operations based on the CPT and ICWGT. To reflect the phenomenon where panel members have different risk attitudes toward different human errors in crane operations, CPT was applied to simulate the psychological behavior of experts. Moreover, after the subjective and objective weights of O, S, and D were determined, we established an optimization model to integrate the different weight results according to the ICWGT. Next, the final risk ranking order for each human error was determined according to the CPT and ICWGT. The remainder of this paper is organized as follows. In Section 2, the CPT and ICWGT are briefly introduced. Section 3 presents the proposed model for human error risk prioritization in crane operations. Section 4 presents a case study of the application of the proposed model. Finally, conclusions are presented in Section 5.

## Materials and methods

### 2.1. Cumulative prospect theory

The CPT [27] proposed by Kahneman and Tversky reflects decision-makers’ subjective attitudes under risk and uncertainty. Consequently, it is used in a variety of decision-making problems in which the risk attitude of the decision-maker is considered. Cheng et al. [29] adopted fuzzy preference relations and CPT to solve the problem of international entry decisions for construction firms. Li and Zhao [30] proposed a safety assessment model based on the CPT and entropy for crane safety grade identification. Li et al. [31] developed a model based on the CPT and the Dempster–Shafer theory for trapezoidal intuitionistic fuzzy MCDM problems.

According to CPT, decision-making processes are based on the overall prospect value, which is expressed as [32]

$$V_{i}=\overset{m}{\underset{j=1}{\sum }}v_{ij}^{+}\pi^{+}(\omega_{j})+\overset{m}{\underset{j=1}{\sum }}v_{ij}^{-}\pi^{-}(\omega_{j}),$$ (1)

where *π*^+^(*ω*_*j*_) and *π*^*—*^(*ω*_*j*_) are decision weight functions, *v*_*ij*_^*+*^ is a positive prospect value, and *v*_*ij*_^*—*^is a negative prospect value. *v*_*ij*_^+^ and *v*_*ij*_^*—*^[32] are calculated as follows:

$$v_{ij}=\{\begin{array}{ll} v_{ij}^{+}={\left(\Delta x_{i}\right)}^{\alpha }, & \Delta x\geq 0\\ v_{ij}^{-}=-\theta {\left(-\Delta x_{i}\right)}^{\beta }, & \Delta x<0 \end{array},$$ (2)

where *α* and *β* are the exponent parameters, which reflect the risk attitudes of decision-makers (*α* = *β* = 0.88) [31]. The parameter *θ* is the risk aversion coefficient (*θ =* 2.25) [31]. △*x*_*i*_ represents the gain or loss between *x*_*i*_ and the reference point *x*_*o*_ [30].

The cumulative prospect weights are determined as follows [32]:

$$\pi (\omega_{j})=\{\begin{array}{l} \pi^{+}(\omega_{j})=\frac{\omega_{j}^{\gamma^{+}}}{{\left[\omega_{j}^{\gamma^{+}}+{\left(1-\omega_{j}\right)}^{\gamma^{+}}\right]}^{\frac{1}{\gamma^{+}}}}\\ \pi^{-}(\omega_{j})=\frac{\omega_{j}^{\gamma -}}{{\left[\omega_{j}^{\gamma -}+{\left(1-\omega_{j}\right)}^{\gamma -}\right]}^{\frac{1}{\gamma -}}} \end{array},$$ (3)

where *ω*_*j*_ is the weight of the risk factor. *γ*^*+*^ and *γ*^*—*^ are risk attitude parameters and are equal to 0.61 and 0.69 [32], respectively.

### 2.2. Improved combination weighting model of game theory (ICWGT)

MCDM aims to support decision-makers in prioritizing alternatives related to many conflicting factors. Determining the weights of the indicators plays an important role in achieving this goal. The relative importance of each indicator is reflected in its weight [33,34]. Generally, weighting methods in MCDM can be divided into three categories: subjective, objective, and combined. In the subjective weighting method, the weights of indicators are determined according to expert knowledge and experience. They are strongly affected by subjective factors, without consideration of information from objective data. In contrast, in the objective weighting method, the weights of the indices are determined using objective information regarding each index. However, objective weights are obtained directly using a mathematical method and do not involve the subjective judgments of experts. Considering the advantages and disadvantages of these two methods, scholars have proposed hybrid weighting methods that combine the strengths of both techniques while avoiding their deficiencies [35]. Research on combination-weighting models is attracting increasing interest.

Combination weighting methods have been proposed, such as the maximizing deviation method [36], the maximizing difference method [34], the relative entropy method [37], the interval estimation method [38], the mathematical programming method [39,40], particle swarm optimization [41], and CWGT [42] The CWGT is one of the most popular theories among these combination weighting models [43] and has been widely applied [44–46]. However, there may be negative values of the weight coefficient after the calculation [47]. This does not satisfy the assumption that all weight coefficients are positive. Although the absolute value approach was proposed to avoid negative values [47], its rationality lacks strict proof. Subsequently, the ICWGT successfully compensated for the insufficiency of the CWGT. The ICWGT procedure [25] is presented below.

Suppose that *L* methods are used to calculate the index weights, and *L* weight vectors are expressed as ***w***_*l*_ = (*w*_*l1*_,*w*_*l2*_,…,*w*_*ln*_) (where *l* = 1, 2, …, *L*, and *n* represents the number of indices). A combination weight vector ***w*** can be established as a linear combination [25]:

$$w=\sum_{l=1}^{L}\alpha_{l}w_{l}^{T}\;(\alpha_{l}>0),$$ (4)

where *α*_*l*_ is the weight combination coefficient.

For obtaining the optimal combination weight vector, the optimization model [25] is expressed as:

$$\left\{ \begin{array}{l} f=\underset{a_{1},a_{2},\ldots ,a_{L}}{\min}\sum_{i=1}^{L}\left|(\sum_{j=1}^{L}a_{j}w_{i}w_{j}^{T})-w_{i}w_{i}^{T}\right|\\ s.t.a_{j}>0,\sum_{j=1}^{L}a_{j}^{2}=1 \end{array}.\right.$$ (5)

Then, the Lagrange function is established as follows:

$$L(a_{j},\lambda )=\sum_{i=1}^{L}\left|(\sum_{j=1}^{L}a_{j}w_{i}w_{j}^{T})-w_{i}w_{i}^{T}\right|+\frac{\lambda }{2}(\sum_{j=1}^{L}a_{j}^{2}-1),$$ (6)

where *λ* represents the Lagrange multiplier.

After the weight coefficient *a*_*j*_ is calculated using Eq (6), the normalized form can be expressed as:

$$a_{j}^{*}=\frac{\sum_{i=1}^{L}w_{i}w_{j}^{T}}{\sum_{j=1}^{L}\sum_{i=1}^{L}w_{i}w_{j}^{T}}.$$ (7)

Finally, the combination weight is:

$$w^{*}=\sum_{j=1}^{L}\alpha_{j}^{*}w_{j}^{T},(j=1,2,\cdots ,L).$$ (8)

## 3. Proposed model for human error risk prioritization in crane operations

In this section, we develop a model that combines the ICWGT and CPT to prioritize human error risks in crane operations. We approach risk evaluation and ranking as an MCDM problem, with risk factors serving as evaluation indices. The proposed model is designed to rank human error risks in crane operations by considering both expert preferences and risk attitudes.

**Fig 2:**
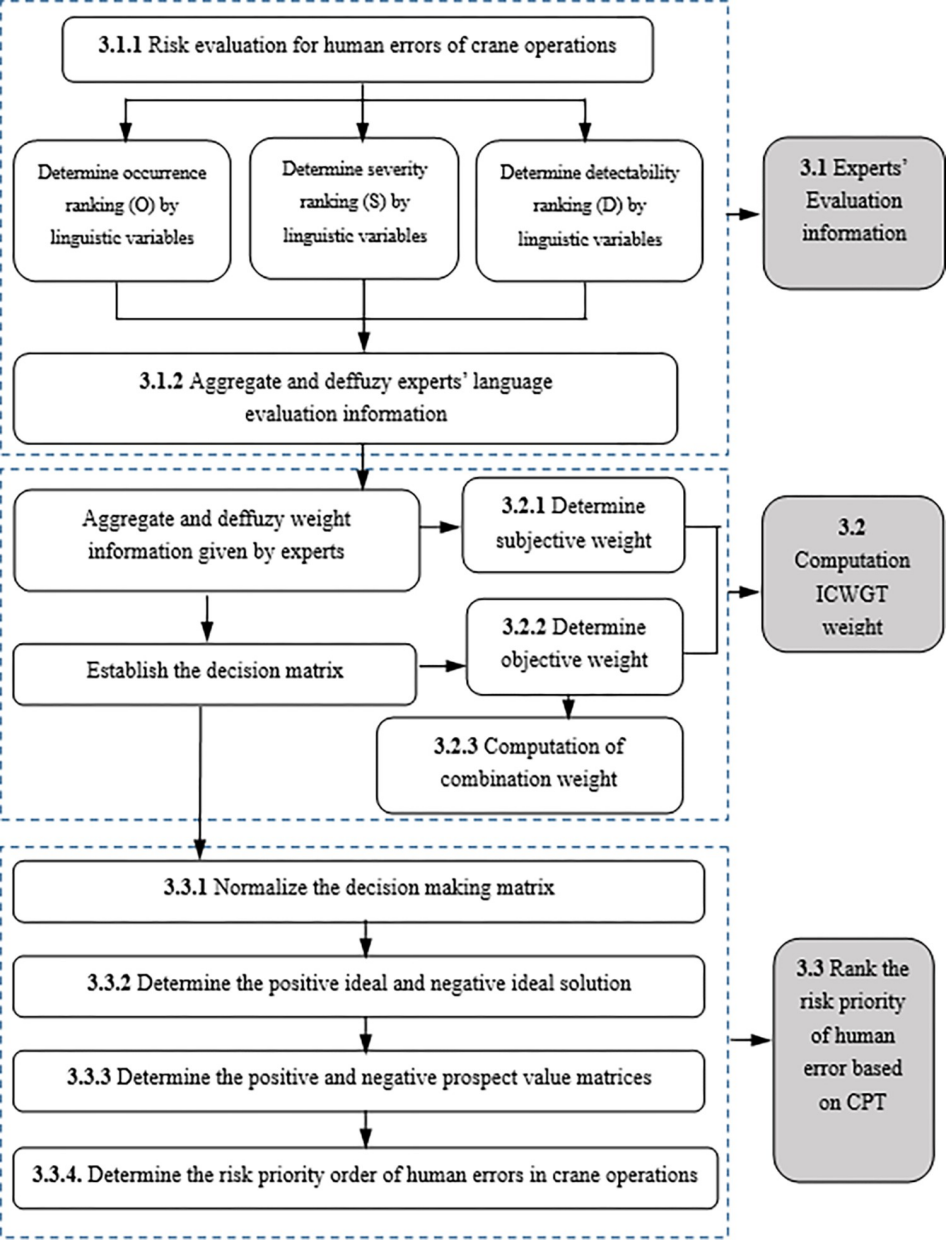
Flowchart of the proposed model.

First, to comprehensively evaluate and quantify the effects of various risk factors related to human error in crane operations, insights and opinions from experts in this field were sought. When reliable and precise numerical data were unavailable, linguistic variables were used to represent expert opinions. Second, given the distinct characteristics of different risk factors, it is essential to assign subjective weights to each risk factor. This is best achieved by consulting industry experts with extensive knowledge and experience in crane operations. In addition to subjective weights, objective weights can be established using entropy. To merge the subjective and objective weights in an effective and meaningful manner, the ICWGT methodology is employed to integrate subjective and objective weights. This methodology ensures that both expert opinions and objective information are considered appropriately, leading to a comprehensive and balanced assessment of the risk of human error in crane operations. Ultimately, the priority of each risk component is determined according to the overall prospect value generated through a comprehensive assessment process. The steps of the proposed approach are shown in Fig 2 and described below.

### 3.1. Experts’ evaluation information

#### 3.1.1. Risk evaluation for human errors of crane operations

We assume that there are *i* failure modes for human error in crane operations, as represented by error no_*i*_ (*i* = 1,2,…, *n*); O, S, and D, which are expressed by *C*_*j*_ (*j* = 1, 2, 3); and *k* assessment experts, as represented by *E*_*k*_ (*k* = 1,…, *K*). Let *e*^(*k*)^_*ij*_ be the evaluation value of error no_*i*_ for index *C*_*j*_ associated with *E*_*k*_. Moreover, the assessment value of each error with respect to the risk factors is expressed as [7]:

$$e_{ij}^{\left(k\right)}=(e_{ij(1)}^{\left(k\right)},\;e_{ij(2)}^{\left(k\right)},\;e_{ij(3)}^{\left(k\right)},\;e_{ij(4)}^{\left(k\right)})\;k=1,2,\cdots ,K,$$ (9)

where *e*^(*k*)^_*ij*(1)_, *e*^(*k*)^_*ij*(2)_, *e*^(*k*)^_*ij*(3)_, and *e*^(*k*)^_*ij*(4)_ represent coordinates of the trapezoidal fuzzy number.

Similarly, the weight vector of the risk factor is expressed as *w*^(*k*)^_*ij*_ *=* (*w*^(*k*)^_*ij*(1)_, *w*^(*k*)^_*ij*(2)_, *w*^(*k*)^_*ij*(3)_, *w*^(*k*)^_*ij*(4)_) for the *k*^th^ expert.

The linguistic variables O, S, and D are presented in Tables 1–3 [7] as trapezoidal fuzzy numbers.

**Table 1:** Linguistic variables for O [7].

Linguistic variable	Error occurrence probability	Fuzzy numbers
Very low	Unlikely	(0,0,1,2)
Low	Very infrequently	(1,2,3,4)
Medium	Occasionally	(3,4,5,6)
High	Repeatedly	(5,6,7,8)
Very high	Inevitably	(7,8,9,10)

**Table 2:** Linguistic variables for S [7].

Linguistic variable	Severity of occurrence	Fuzzy numbers
Very low	No effect	(0,0,1,2)
Low	Very slight	(1,2,3,4)
Medium	Moderate	(3,4,5,6)
High	Serious	(5,6,7,8)
Very high	Hazardous	(7,8,9,10)

**Table 3:** Linguistic variables for D [7].

Linguistic variable	Error detection ability	Fuzzy numbers
AC	Almost certain	(0,0,1,2)
H	Very easy	(1,2,3,4)
M	Moderately easy	(3,4,5,6)
L	Very difficult	(5,6,7,8)
AI	Almost impossible	(7,8,9,10)

#### 3.1.2. Aggregation of experts’ language evaluation information

For each expert, the integrated risk score is given as follows [7]:

$$Agg(e_{ij})=Agg({e^{\left(1\right)}}_{ij},{e^{\left(2\right)}}_{ij},\ldots ,{e^{\left(k\right)}}_{ij}),$$ (10)

where Agg (^.^) represents the aggregation function.


$$e_{ij(1)}^{\left(k\right)}=\underset{k}{\min}(e_{ij(1)}^{\left(k\right)})$$ (11)

$$e_{ij(2)}^{\left(k\right)}=\frac{1}{K}\sum_{k=1}^{K}\left(e_{ij(2)}^{\left(k\right)}\right)$$ (12)

$$e_{ij(3)}^{\left(k\right)}=\frac{1}{K}\sum_{k=1}^{K}\left(e_{ij(3)}^{\left(k\right)}\right)$$ (13)

$$e_{ij(4)}^{\left(k\right)}=\underset{k}{\max}(e_{ij(4)}^{\left(k\right)})$$ (14)

The subjective weights provided by the different experts can be aggregated in the same manner. The centroid defuzzification of trapezoidal fuzzy numbers is given by the following Equ [7]:

$$e_{ij}=\frac{1}{3}[e_{ij(1)}+e_{ij(2)}+e_{ij(3)}+e_{ij(4)}-\frac{\left\{e_{ij(3)}\cdot e_{ij(4)}-e_{ij(1)}\cdot e_{ij(2)}\right\}}{\left\{\left(e_{ij(3)}+e_{ij(4)})-(e_{ij(1)}+e_{ij(2)}\right)\right\}}].$$ (15)

### 3.2. Computation ICWGT weight

#### 3.2.1. Determination of subjective weights based on experts’ evaluation information

With regard to the characteristics of risk factors, subjective weights were determined according to expert experience. Similarly, using the method presented in Section 3.1.1, the weight of the risk factor is expressed as *w*^(*k*)^_*ij*_ *=* (*w*^(*k*)^_*ij*(1)_, *w*^(*k*)^_*ij*(2)_, *w*^(*k*)^_*ij*(3)_, *w*^(*k*)^_*ij*(4)_) for the *k*^th^ expert. The subjective weights provided by different experts can be aggregated and defuzzified using this method (see Section 3.1.2). The linguistic variables of the weights of the risk factors are presented in Table 4 [7] as trapezoidal fuzzy numbers.

**Table 4:** Linguistic variables for the weights of risk factors [7].

Linguistic variables	Fuzzy numbers
None (N)	(0,0,1,2)
Low (L)	(1,2,3,4)
Moderate (M)	(3,4,5,6)
High (H)	(5,6,7,8)
Very high (VH)	(7,8,9,10)

#### 3.2.2. Determination of objective weights based on entropy

Step 1: Establish decision matrix

This process transforms the defuzzified trapezoidal fuzzy information into a decision vector. Consequently, the decision matrix for human error is obtained as follows:

$$\begin{array}{l} e=[e_{ij}]=\left[\begin{array}{cccc} e_{11} & e_{12} & \cdots & e_{1j}\\ e_{21} & e_{22} & \cdots & e_{2j}\\ \vdots & \vdots & \vdots & \vdots \\ e_{n1} & e_{n2} & \cdots & e_{nj} \end{array}\right]\\ \;i=1,2,\cdots ,\;n;\;j=1,2,3 \end{array},$$ (16)

where *e*_*ij*_ denotes the defuzzified trapezoidal fuzzy information of the *j*^th^ risk factor with respect to error no_*i*_.

Step 2: Determine entropy weight of risk factors

After the decision matrix of the failure modes is obtained, the weights O, S, and D are determined according to the entropy weight method. The entropy weight is calculated as follows [48]:

The information entropy of each risk factor is expressed as:

$$E_{j}=‐\frac{\sum_{i=1}^{n}\mu_{ji}\ln\mu_{ji}}{\ln n},j=1,2,3.,$$ (17)

where *μ*_*ji*_ denotes the projection value of human error evaluation information. To ensure that *μ*_*ji*_ln*μ*_*ji*_ has mathematical meaning, *μ*_*ji*_ln*μ*_*ji*_ is defined as 0 when *μ*_*ji*_ *=* 0. *μ*_*ji*_ can be calculated as:

$$\mu_{ji}=e_{ji}/\sum_{i=1}^{n}e_{ji}.$$ (18)

The entropy weight of each risk factor is expressed as:

$$w_{oj}=\frac{1-E_{j}}{3-\sum_{j=1}^{3}E_{j}},(0\leq w_{oj}\leq 1,\sum_{j=1}^{3}w_{oj}=1).$$ (19)

#### 3.2.3. Computation of combination weight

Let ***w***_***1***_ and ***w***_***2***_ denote the subjective and objective weight vectors, respectively. The combination weight calculated via the ICWGT is denoted as ***w***_(ICWGT)_, which is combined using Eqs (7) and (8).

### 3.3. Rank risk priority of human error based on CPT

#### 3.3.1. Normalize decision-making matrix

To better reflect the gains and losses in prospect theory, it is necessary to normalize the decision-making matrix ***e*** = (*e*_*ij*_). The normalized values of the benefit- and cost-related indices were calculated as follows [49]:

For the cost index, *e*_*ij*_ can be normalized as:

$$r_{ij}=\frac{\underset{j}{\max}(e_{ij})-e_{ij}}{\underset{j}{\max}(e_{ij})-\underset{j}{\min}(e_{ij})},\;i=1,2,\cdots ,n;j=1,2,3.$$ (20)

For the beneficial index, *e*_*ij*_ can be normalized as:

$$r_{ij}=\frac{e_{ij}-\underset{j}{\min}(e_{ij})}{\underset{j}{\max}(e_{ij})-\underset{j}{\min}(e_{ij})},\;i=1,2,\cdots ,n;j=1,2,3.$$ (21)

Here, $\underset{j}{\max}(e_{ij})$ represents the maximum performance rating among the human errors for the *i*^th^ risk factor, and $\underset{j}{\min}(e_{ij})$ represents the minimum performance rating among the human errors for the *i*^th^ risk factor.

The normalized decision-making matrix ***R*** is expressed as:

$$\begin{array}{l} R=[r_{ij}]=\left[\begin{array}{cccc} r_{11} & r_{12} & \cdots & r_{1j}\\ r_{21} & r_{22} & \cdots & r_{2j}\\ \vdots & \vdots & \vdots & \vdots \\ r_{n1} & r_{n2} & \cdots & r_{nj} \end{array}\right]\\ \;i=1,2,\cdots ,n:j=1,2,3 \end{array}.$$ (22)

In this study, risk factors (O, S, and D) were used as cost indices [50].

#### 3.3.2. Determine positive ideal and negative ideal solutions

When making a decision based on prospect theory, decision-makers typically evaluate the gains and losses of alternatives according to reference points. In this study, the positive ideal solution (PIS) and negative ideal solution (NIS) were considered as the reference points [51–53]. The PIS is denoted as ***S***^*+*^ *=* (*r*_1_^*+*^, *r*_2_^*+*^, …, *r*_*m*_^*+*^), and the NIS is denoted as ***S***^−^ *=* (*r*_1_^*-*^, *r*_2_^*-*^, *…*, *r*_*m*_^*-*^).

#### 3.3.3 Determine positive and negative prospect value matrices

According to matrix ***R***, the PIS is denoted as ***S***^*+*^ = (*r*_1_^*+*^, *r*_2_^*+*^, *…*, *r*_*m*_^*+*^), and the NIS is denoted as ***S***^−^ = (*r*_1_^*-*^, *r*_2_^*-*^, *…*, *r*_*m*_^*-*^). Using Eq (2), the positive prospect value matrix is established as:

$$V^{+}={\left(v_{ij}^{+}\right)}_{n\times m}=[\begin{array}{cccc} v_{11}^{+} & v_{12}^{+} & \cdots & v_{1m}^{+}\\ v_{21}^{+} & v_{22}^{+} & \cdots & v_{2m}^{+}\\ \vdots & \vdots & \vdots & \vdots \\ v_{n1}^{+} & v_{n2}^{+} & \cdots & v_{nm}^{+} \end{array}],$$ (23)

where *i* = 1, 2, …, *n*; *j* = 1, 2, …, *m*; and *v*_*ij*_^+^ = (*r*_*ij*_*−r*_*j*_^*-*^)^0.88^.

The negative prospect value matrix is obtained as:

$$V^{-}={\left(v_{ij}^{-}\right)}_{n\times m}=[\begin{array}{cccc} v_{11}^{-} & v_{12}^{-} & \cdots & v_{1m}^{-}\\ v_{21}^{-} & v_{22}^{-} & \cdots & v_{2m}^{-}\\ \vdots & \vdots & \vdots & \vdots \\ v_{n1}^{-} & v_{n2}^{-} & \cdots & v_{nm}^{-} \end{array}],$$ (24)

where *i* = 1, 2, …, *n*; *j* = 1, 2, …, *m*; and *v*_*ij*_^-^ = *–*2.25(*r*_*j*_^*+*^–*r*_*ij*_)^0.88^.

#### 3.3.4. Determine risk priority order of human errors in crane operations

The integrated prospect value *V*_*i*_ for the failure modes of human error was calculated using Eq (1). Human error risk prioritization was calculated by ranking *V*_*i*_ for each error.

## 4. Case study

The proposed model was applied to prioritize human error risk in overhead crane operations. Next, we conducted a comparative study and sensitivity analysis to verify the effectiveness of the method described above. The results of the risk prioritization provide valuable guidance for safety management departments. The proposed model framework is implemented for human error risk prioritization in crane operations using the following steps:

### 4.1. Determine human errors and collect experts’ evaluation data

Overhead cranes are widely used in industrial and mining enterprises and can easily fail because of human error during operations. The determination of human errors during overhead crane operations plays an important role in the proposed model. As shown in Table 5, 21 human errors were identified [7], which are denoted as error no_*i*_ (*i* = 1, 2, …, 21). Each error was evaluated according to the three risk factors (O, S, and D) presented in Tables 1–3 [7]. In this case, four experts E_1_, E_2_, E_3_, E_4_ evaluated the human errors with regard to O, S, and D. The evaluation results for each error with regard to O, S, and D and the weights of O, S, and D are presented in Table 6 [7].

**Table 5:** Human error components selected for risk prioritization [7].

Error no	Human error descriptions
1	Check omitted/incomplete (tracks of obstacles and damage)
2	Check omitted/incomplete (wheels, flanges, park brakes and gears for lube and damage, wheel motors over heating)
3	Check omitted/incomplete (damaged wiring)
4	Check omitted/incomplete (joints)
5	Check omitted/incomplete (electrical controls and operating buttons)
6	Check omitted (loose objects)
7	Check omitted/incomplete (rails and wheels of trolley and bridge for wear and cracks)
8	Check omitted/incomplete (hoist machinery and rope for damage or other fault)
9	Check omitted (bridge)
10	Check omitted/incomplete (sling and all other lifting accessories for damage)
11	Check omitted/incomplete (sling and all other lifting accessories for load limit)
12	Check omitted (load limit of hoist rope)
13	Misalign (the beam is not properly tied to the hook while loading)
14	Misalign (Belt is not tied around the hardware (load) properly)
15	Misalign (Hardware is not attached to beam by belt properly)
16	Wrong selection made (Wrong pendant push button pressed while operating the crane)
17	Misalign (Hardware not smoothly placed on the vehicle)
18	Information not obtained (Information about the position to lower the hardware not received)
19	Information not communicated (Information about the position to lower the hardware not communicated)
20	Wrong information obtained (Wrong information about the position to lower the hardware received)
21	Wrong information communicated (Wrong information about the position to lower the hardware communicated)

**Table 6:** Experts’ judgment of each human error [7].

Error no	E_1_						E_2_			E_3_			E_4_		
	O	S		D		O		S	D	O	S	D	O	S	D
1	L	L		AC		M		M	AC	M	H	AC	M	H	AC
2	L	L		AC		L		M	L	M	H	H	M	VH	H
3	M	M		H		M		M	M	L	VH	H	M	H	H
4	L	H		H		L		H	M	L	VH	M	L	VH	H
5	L	H		H		L		VH	M	L	VH	M	L	VH	H
6	L	L		AC		VH		VH	M	M	H	M	L	H	H
7	M	M		M		M		H	L	M	H	M	M	H	H
8	L	H		H		L		VH	H	L	H	M	L	VH	H
9	L	H		H		L		VH	H	L	VH	M	M	VH	M
10	M	L		AC		H		VH	M	M	VH	L	L	VH	H
11	M	VH		M		M		VH	M	H	VH	L	L	VH	H
12	M	M		H		M		VH	AC	H	VH	L	M	VH	H
13	M	H		M		M		VH	M	L	H	M	M	VH	H
14	M	H		M		M		VH	M	L	H	M	M	VH	H
15	H	VH		M		M		VH	M	L	H	M	M	VH	H
16	H	L		AC		VH		VH	L	M	M	H	L	VH	AC
17	L	L		L		M		L	AC	M	H	H	L	H	H
18	L	M		AC		M		H	AC	M	M	H	L	H	AC
19	L	H		AC		M		H	AC	L	M	H	L	H	AC
20	M	H		AC		M		H	AC	L	M	H	L	H	AC
21	M	M		AC		M		H	AC	L	M	H	L	H	AC
Subjective Weight	H	VH		M		H		H	M	M	H	M	VH	H	H

### 4.2. Integration and defuzzification of evaluation information of human error

To rank the risk prioritization of human errors, the experts’ linguistic evaluation information for O, S, and D is integrated, and their weights are converted into trapezoidal fuzzy numbers using Eqs (11)–(14) and then defuzzified to crisp values using Eq (15). The results of the integration and defuzzification are presented in Table 7 [7].

**Table 7:** Results of integration and defuzzification [7].

Error no.	O		S		D	
	Fuzzy	crisp	Fuzzy	crisp	Fuzzy	crisp
1	(1,3.5,4.5,6)	3.69	(1,4.5,5.5,8)	4.68	(0,0,1,2)	0.77
2	(1,3,4,6)	3.50	(1,5,6,10)	5.50	(0,2.5,3.5,8)	3.62
3	(1,3.5,4.5,6)	3.69	(3,5.5,6.5,10)	6.31	(1,2.5,3.5,6)	3.30
4	(1,2,3,4)	2.50	(5,7,8,10)	7.50	(1,3,4,6)	3.50
5	(1,2,3,4)	2.50	(5,7,5,8.5,10)	7.69	(1,3,4,6)	3.50
6	(1,4,5,10)	5.13	(1,5.5,6.5,10)	5.68	(0,2.5,3.5,6)	3.00
7	(3,4,5,6)	4.50	(3,6,7,8)	6.50	(1,4,5,8)	4.50
8	(1,2,3,4)	2.50	(5,7,8,10)	7.50	(1,2.5,3.5,6)	3.30
9	(1,2.5,3.5,6)	3.30	(5,7.5,8.5,10)	7.69	(1,3,4,6)	3.50
10	(1,4,5,8)	4.50	(1,6.5,7.5,10)	6.05	(0,3,4,6)	3.81
11	(1,4,5,8)	4.50	(7,8,9,10)	8.50	(1,4,5,8)	4.50
12	(3,5,6,8)	5.50	(3,7,8,10)	6.87	(0,2.5,3.5,8)	3.62
13	(1,3.5,4.5,6)	3.69	(5,7,8,10)	7.50	(1,3.5,4.5,6)	3.69
14	(1,3.5,4.5,6)	3.69	(5,7,8,10)	7.50	(1,3.5,4.5,6)	3.69
15	(1,4,5,8)	4.50	(5,7.5,8.5,10)	7.69	(1,3.5,4.5,6)	3.69
16	(1,4.5,5.5,10)	5.31	(1,5.5,6.5,10)	5.68	(0,2,3,8)	3.44
17	(1,3,4,6)	3.50	(1,4,5,8)	4.50	(0,2.5,3.5,8)	3.62
18	(1,3,4,6)	3.50	(3,5,6,8)	5.50	(0,0.5,1.5,4)	1.60
19	(1,2.5,3.5,6)	3.30	(3,5.5,6.5,8)	5.69	(0,0.5,1.5,4)	1.60
20	(1,3,4,6)	3.50	(3,5.5,6.5,8)	5.69	(0,0.5,1.5,4)	1.60
21	(1,3,4,6)	3.50	(3,5,6,8)	5.50	(0,0.5,1.5,4)	1.60
Subjective weight	(0.3,0.6,0.7,1)	0.65	(0.5,0.65,0.75,1)	0.73	(0.3,0.45,0.55,0.8)	0.53

### 4.3. Calculation of combination weights

**Fig 3:**
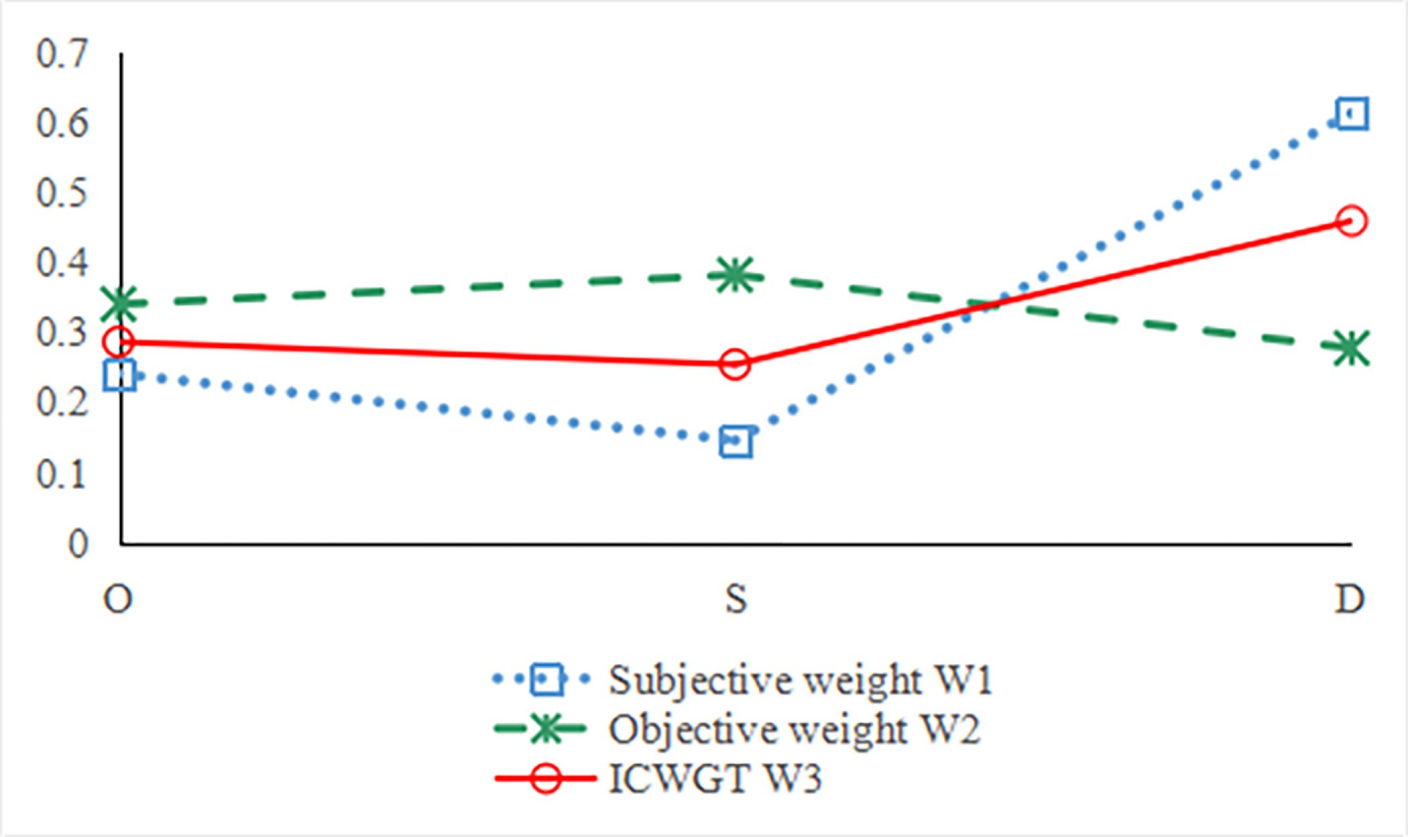
Calculation results of various weighting methods.

To reflect the potential information of evaluation data, the entropy weight method is adopted to calculate the weights of O, S, and D. According to Table 7, *μ*_*ji*_ can be obtained, as shown in Table 8. Considering the data provided in Table 7, Eqs (17) and (19) are used to determine the objective weight. The normalized subjective weights of O, S, and D are then calculated, and they are presented in Table 9. Next, the integrated weights are calculated using Eqs (7) and (8) according to the ICWGT. The values for the three different types of weights (O, S, and D) are presented in Table 9. Fig 3 shows that the integrated weights calculated via the ICWGT are the optimal equilibrium values between the other two.

**Table 8:** Projection values of human error evaluation information.

Error no.	O	S	D
1	0.0460	0.0345	0.0118
2	0.0436	0.0405	0.0553
3	0.0460	0.0465	0.0504
4	0.0311	0.0553	0.0535
5	0.0311	0.0567	0.0535
6	0.0639	0.0419	0.0458
7	0.0560	0.0479	0.0688
8	0.0311	0.0553	0.0504
9	0.0411	0.0567	0.0535
10	0.0560	0.0446	0.0582
11	0.0560	0.0626	0.0688
12	0.0685	0.0506	0.0553
13	0.0460	0.0553	0.0564
14	0.0460	0.0553	0.0564
15	0.0560	0.0567	0.0564
16	0.0661	0.0419	0.0526
17	0.0436	0.0332	0.0553
18	0.0436	0.0405	0.0244
19	0.0411	0.0419	0.0244
20	0.0436	0.0419	0.0244
21	0.0436	0.0405	0.0244

**Table 9:** Weights of risk factors.

risk factor	Subjective weight *W*_1_	Objective weight *W*_2_	ICWGT *W*_3_
O	0.3403	0.2408	0.2865
S	0.3822	0.1459	0.2543
D	0.2775	0.6133	0.4593

### 4.4. Determination of positive and negative prospect matrices

According to Table 8 and Eqs (20) and (21), the normalized decision matrix is obtained as:

$$R=[\begin{array}{ccc} 0.6033 & 0.9550 & 1\\ 0.6667 & 0.7500 & 0.2359\\ 0.6033 & 0.5475 & 0.3217\\ 1 & 0.2500 & 0.2681\\ 1 & 0.2025 & 0.2681\\ 0.1233 & 0.7050 & 0.4021\\ 0.3333 & 0.5000 & 0\\ 1 & 0.2500 & 0.3217\\ 0.7333 & 0.2025 & 0.2681\\ 0.3333 & 0.6125 & 0.1850\\ 0.3333 & 0 & 0\\ 0 & 0.4075 & 0.2359\\ 0.6033 & 0.2500 & 0.2172\\ 0.6033 & 0.2500 & 0.2172\\ 0.3333 & 0.2050 & 0.2172\\ 0.0633 & 0.7050 & 0.2842\\ 0.6667 & 1 & 0.2359\\ 0.6667 & 0.7500 & 0.7775\\ 0.7333 & 0.7025 & 0.7775\\ 0.6667 & 0.7025 & 0.7775\\ 0.6667 & 0.7500 & 0.7775 \end{array}].$$

From matrix ***R***, the PIS and NIS are determined as follows:

$$S^{+}=(1,1,1);S^{-}=(0,0,0).$$

Using Eqs (23) and (24), the positive and negative prospect matrices are determined. The results are presented below.

$$V^{-}=[\begin{array}{ccc} -0.9972 & -0.1469 & 0\\ -0.8557 & -0.6643 & -1.7756\\ -0.9972 & -1.1198 & -1.5989\\ 0 & -1.7468 & -1.7096\\ 0 & -1.8438 & -1.7096\\ -2.0039 & -0.7685 & -1.4308\\ -1.5748 & -1.2226 & -2.25\\ 0 & -1.7468 & -1.5989\\ -0.7031 & -1.8438 & -1.7096\\ -1.5748 & -0.9769 & -1.8793\\ -2.25 & -1.4195 & -1.7756\\ -0.9972 & -1.7468 & -1.8139\\ -0.9972 & -1.7468 & -1.8139\\ -1.5748 & -1.8438 & -1.8139\\ -2.1241 & -0.7685 & -1.6765\\ -0.8557 & 0 & -1.7756\\ -0.8557 & -0.6643 & -0.5996\\ -0.7031 & -0.7742 & -0.5996\\ -0.8557 & -0.7742 & -0.5996\\ -0.8557 & -0.6643 & -0.5996 \end{array}]\;V^{+}=[\begin{array}{ccc} 0.6410 & 0.9603 & 1\\ 0.6999 & 0.7763 & 0.2806\\ 0.6410 & 0.5885 & 0.3686\\ 1 & 0.2952 & 0.3140\\ 1 & 0.2453 & 0.3140\\ 0.1585 & 0.7352 & 0.4486\\ 0.3803 & 0.5434 & 0\\ 1 & 0.2952 & 0.3686\\ 0.7611 & 0.2453 & 0.3140\\ 0.3803 & 0.6496 & 0.2265\\ 0.3803 & 0 & 0\\ 0 & 0.4538 & 0.2806\\ 0.6410 & 0.2952 & 0.2608\\ 0.6410 & 0.2952 & 0.2608\\ 0.3803 & 0.2453 & 0.2608\\ 0.0882 & 0.7352 & 0.3305\\ 0.6999 & 1 & 0.2806\\ 0.6999 & 0.7763 & 0.8013\\ 0.7611 & 0.7329 & 0.8013\\ 0.6999 & 0.7329 & 0.8013\\ 0.6999 & 0.7763 & 0.8013 \end{array}]$$

### 4.5. Rank human error risk prioritization in overhead crane operations

**Fig 4:**
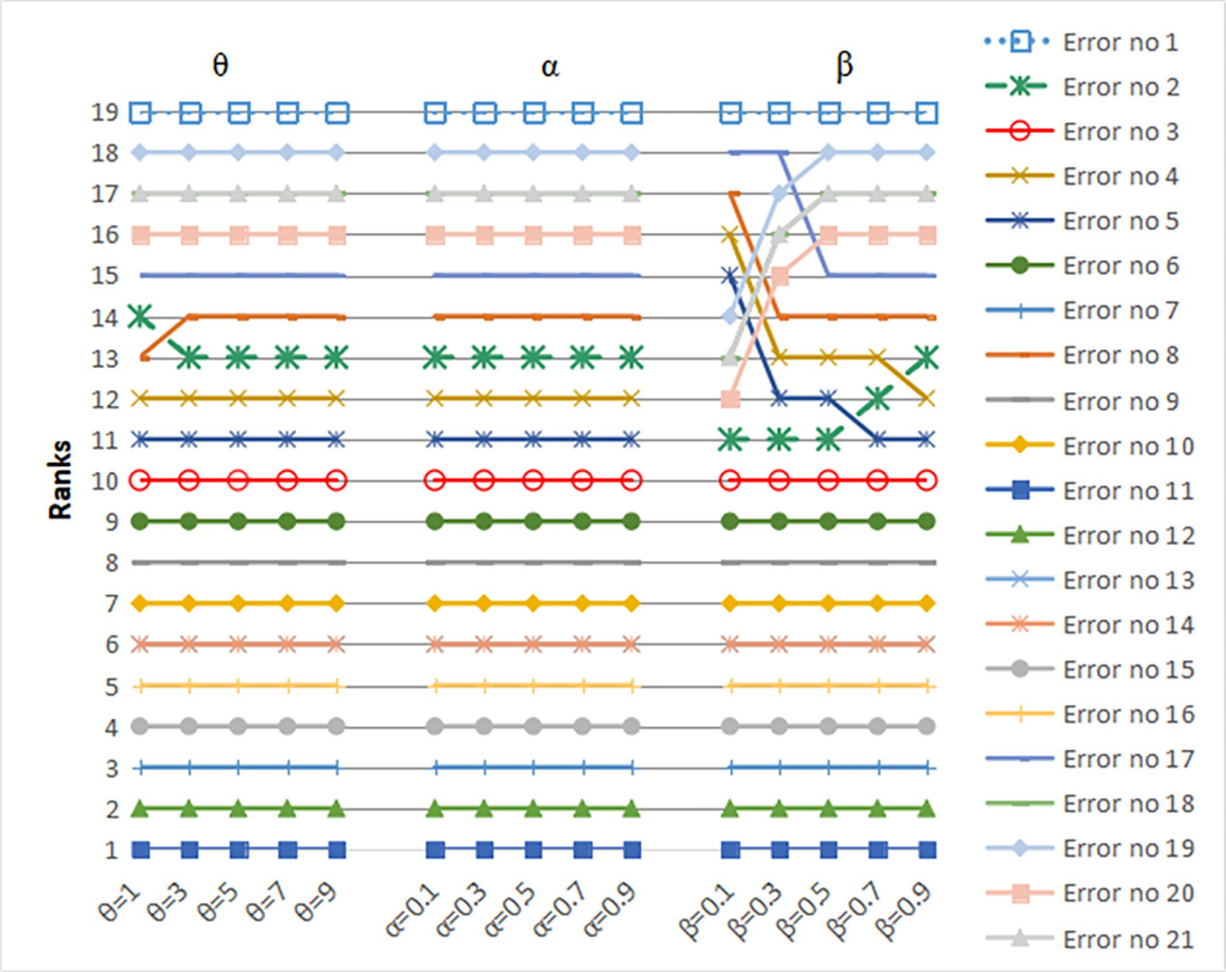
Sensitivity analysis.

**Fig 5:**
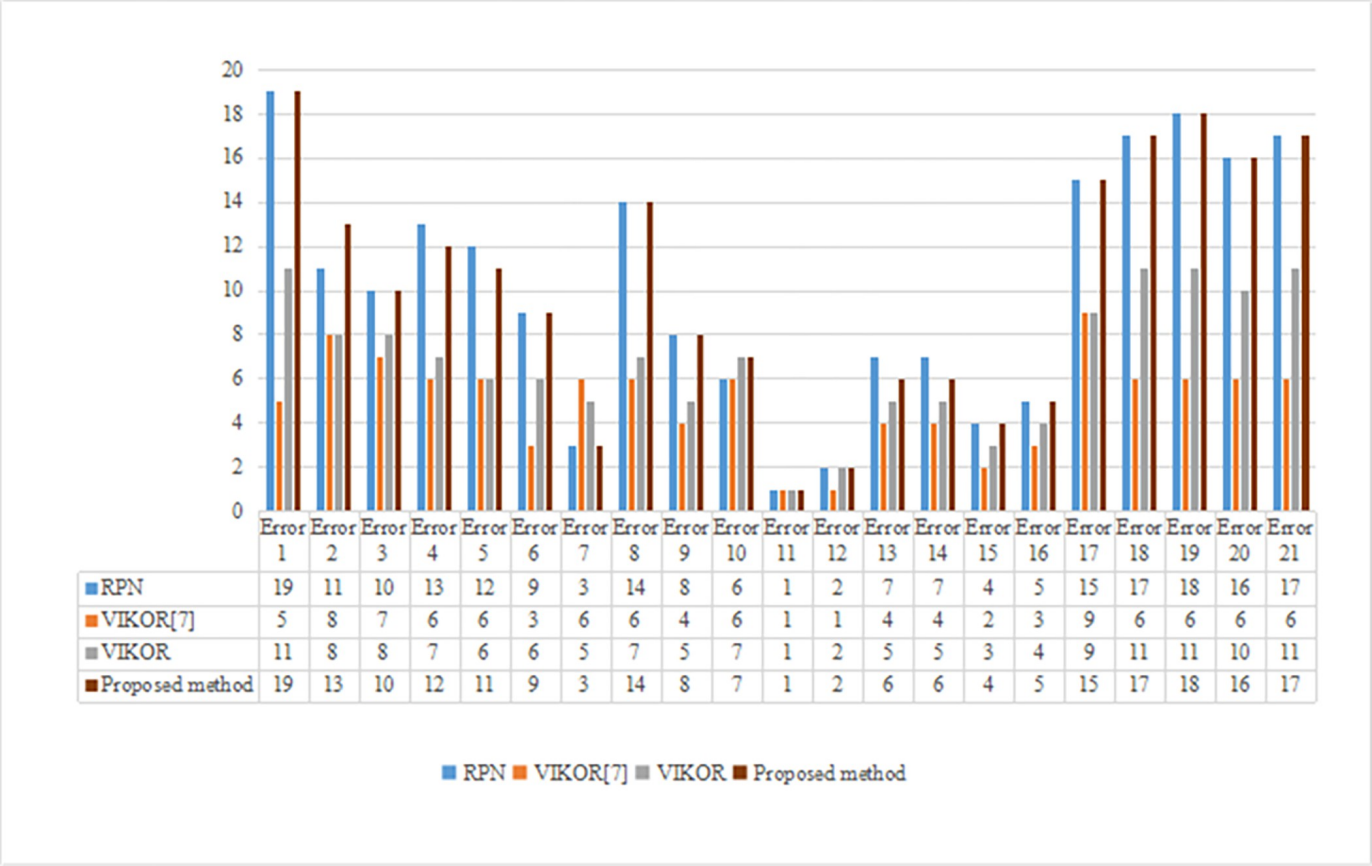
Comparison of risk priority ranking results of different methods.

According to the overall prospect values calculated using Eq (5), the risk prioritization of all the errors is as follows: error 11 > error 12> error 7 > error 15 > error 16 > error 13 = error 14 > error 10 > error 9 > error 6 > error 3 > error 5 > error 4 > error 2 > error 8 > error 17 > error 20 > error 18 = error 21 > error 19 > error 1. Clearly, error 11 has the highest risk priority and deserves focus on preventive measures, whereas error 1 has the lowest relative risk. The ranking of the errors is presented in Table 10. This can help risk managers identify the key points in overhead crane safety management and would be advantageous in formulating reasonable suggestions. Comparative and sensitivity analyses were performed, and the results are shown in Figs 4 and 5, respectively.

**Table 10:** Ranking results of the errors obtained using different methods.

Error no	Subjective weight				Subjective weight		ICWGT	
	VIKOR[7] (Value of Q)	Risk Rank	RPN	Risk Rank	VIKOR (Value of Q)	Risk Rank	Proposed method (V_*i*_)	Risk Rank
1	0.6753	5	13.30	19	0.0401	11	0.5196	19
2	0.3704	8	69.69	11	0.3841	8	-0.6730	13
3	0.5445	7	76.84	10	0.3880	8	-0.8156	10
4	0.6185	6	65.63	13	0.5540	7	-0.7275	12
5	0.6532	6	67.29	12	0.5999	6	-0.7709	11
6	0.7841	3	87.42	9	0.6334	6	-1.0351	9
7	0.6158	6	131.63	3	0.6964	5	-1.5508	3
8	0.6328	6	61.88	14	0.5436	7	-0.6582	14
9	0.7399	4	88.82	8	0.6609	5	-1.0691	8
10	0.6238	6	103.73	6	0.5327	7	-1.1974	7
11	0.9467	1	172.13	1	1.0000	1	-2.0148	1
12	0.9288	1	136.78	2	0.8481	2	-1.6533	2
13	0.7340	4	102.12	7	0.6544	5	-1.2227	6
14	0.7340	4	102.12	7	0.6544	5	-1.2227	6
15	0.8564	2	127.69	4	0.7621	3	-1.5312	4
16	0.7918	3	103.75	5	0.7079	4	-1.2478	5
17	0.2792	9	57.02	15	0.3200	9	-0.4105	15
18	0.6116	6	30.80	17	0.0811	11	0.0393	17
19	0.6073	6	30.04	18	0.0786	11	0.0616	18
20	0.6290	6	31.86	16	0.0938	10	-0.0061	16
21	0.6116	6	30.80	17	0.0811	11	0.0393	17

### 4.6. Sensitivity analysis

To verify the robustness of the proposed model and examine the influence of associated parameters on human error risk prioritization in overhead crane operations, a sensitivity analysis was conducted using the Kingsoft Office software WPS. This method involves examining a single parameter at a time while assuming that θ, α, and β are independent of each other; that is, only one parameter is changed at a time, while the other parameters remain constant, to analyze its influence on the results. Using the WPS software, various scenarios and simulations can be performed to determine how changes in the parameters affect the overall outcome. In view of the CPT, the risk preferences for determining the risk prioritization are reflected by the parameters θ, α, and β in Eq (1). The value of θ (risk aversion coefficient) is >1. For a higher prospect value, the decision-maker is more sensitive to risk. In the gain interval, α represents the degree of concavity of the value function. In the loss interval, β represents the degree of convexity of the value function. For higher values of α and β, the decision-maker is less likely to avoid risk. To analyze the effect of each risk preference coefficient on the decision outcomes, we established three dynamic scenarios [54] by systematically adjusting the risk parameters. (1) In dynamic scenario I, the coefficients α and β remained constant while the parameter θ was varied from 1 to 10. (2) In dynamic scenario II, the coefficients θ and β remained constant while the parameter α was varied from 0 to 1. (3) In dynamic scenario III, the coefficients θ and α remained constant while the parameter β was varied from 0 to 1.

#### 4.6.1. Control parameter θ

Taking θ = 1, 3, 5, 7, and 9, respectively, with the other parameters constant, the results of human error risk prioritization are presented in Fig 4. As shown, changes in the risk aversion coefficient θ affect the risk prioritization of human errors. The results fluctuated for errors 2 and 8, indicating that they are vulnerable to the influence of θ. However, other error ranks remained unchanged. For example, errors 11 and 1 maintained the highest and lowest risk, respectively. Thus, the general trend of risk prioritization remained nearly unchanged, with high rank stability.

#### 4.6.2. Control parameter α

A sensitivity analysis was conducted with α = 0.1, 0.3, 0.5, 0.7, and 0.9. The calculation results are presented in Fig 4. The results are consistent with our previous findings. The errors were ranked as follows: error 11 > error 12 > error 7 > error 15 > error 16 > error 13 = error 14 > error 10 > error 9 > error 6 > error 3 > error 5 > error 4 > error 2 > error 8 > error 17 > error 20 > error 18 = error 21 > error 19 > error 1. Our results indicated that the parameter α did not affect the results for the ranking order of the 21 errors, as shown in Fig 4. Clearly, the rankings of human errors are not susceptible to variations in α.

#### 4.6.3. Control parameter *β*

The ranking results obtained with *β* varying from 0.1 to 0.9 are shown in Fig 4. The results fluctuated for errors 2, 20, 21, 18, 19, 5, 4, 8, and 17, indicating that they are vulnerable to the influence of the parameter *β*. As shown in Fig 4, error 11 maintained the highest risk priority with changes in *β*. For example, with *β =* 0.1, the ranking of error risk was as follows: error 11 > error 12 > error 7 > error 15 > error 16 > error 13 = error 14 > error 10 > error 9 > error 6 > error 3 > error 2 > error 20 > error 18 = error 21 > error 19 > error 5 > error 4 > error 8 > error 17 > error 1. When *β* was 0.5, the ranking was as follows: error 11 > error 12 > error 7 > error 15 > error 16 > error 13 = error 14 > error 10 > error 9 > error 6 > error 3 > error 2 > error 5 > error 4 > error 8 > error 17 > error 20 > error 18 = error 21 > error 19 > error 1. When *β* was 0.9, the ranking was as follows: error 11 > error 12 > error 7 > error 15 > error 16 > error 13 = error 14 > error 10 > error 9 > error 6 > error 3 > error 5 > error 4 > error 2 > error 8 > error 17 > error 20 > error 18 = error 21 > error 19 > error 1.

The other human error risk rankings remained the same, indicating that the proposed framework is relatively stable.

### 4.7 Comparison analysis

The first comparative analysis was conducted between the proposed method and the RPN of traditional FMEA. When the RPN was used to rank the risk prioritization of human error in overhead crane operations, the risk order of the 21 human errors was as follows: error 11 > error 12 > error 7 > error 15 > error 16 > error 10 > error 13 = error 14 > error 9 > error 6 > error 3 > error 2 > error 5 > error 4 > error 8 > error 17 > error 20 > error 18 = error 21 > error 19 > error 1. The order obtained via the proposed method was as follows: error 11 > error 12 > error 7 > error 15 > error 16 > error 13 = error 14 > error 10 > error 9 > error 6 > error 3 > error 5 > error 4 > error 2 > error 8 > error 17 > error 20 > error 18 = error 21 > error 19 > error 1. As shown in Fig 5, the risk prioritizations of errors 2, 4, 5, 10, and 13 were not consistent between the RPN and the proposed method, whereas the other prioritizations were the same. The main reason for this is that the proposed method can address the disadvantages of the RPN of traditional FMEA mentioned in Section 1. The proposed method ranks human error risk prioritization according to overall prospect values. In addition, the ICWGT was applied to the proposed FMEA framework by considering the rationality of the risk-factor weight aggregation. In this case study, the proposed method and RPN produced similar ranking results, indicating the effectiveness of these approaches. However, it is important to note that use of the RPN may not always be suitable in practical scenarios. For instance, let us consider the representation of errors as (O = 6, S = 5, D = 7), (O = 6, S = 7, D = 5), and (O = 5, S = 6, D = 7), all of which have RPN scores of 210. It is evident that relying solely on RPN scores does not offer a meaningful basis for comparing the risks associated with these errors. In contrast, the proposed method, which incorporates weights and considers the decision-makers’ risk attitudes, can effectively address such situations. Thus, the risk prioritization determined via the proposed method has advantages over the RPN of traditional FMEA. To further verify the applicability and efficiency of the proposed model, another comparative analysis was performed using Mandal’s method [7] and VIKOR. The results are shown in Fig 5.

The second comparison was conducted using Mandal’s study [7], in which the VIKOR technique was applied to the aforementioned case. We also ranked the 21 errors using VIKOR. From Table 10 and Fig 5, the value of *Q*_*i*_ was significantly different from that reported in the literature [7]. For example, if O and S are taken as cost indices and D is taken as a benefit index, the risk priority index *Q*_1_ for Error 1 is calculated using VIKOR as follows:

$$Q_{1}=v(\frac{S_{1}-S*}{S^{-}-S*})+(1-v)(\frac{R_{1}-R*}{R^{-}-R*}),$$

where S* = 0.341707, *S*^−^ *=* 1.207565, R* = 0.216667, *R*^−^ *=* 0.73, *v* = 0.5, *S*_1_ = 0.820683, and *R*_1_ = 0.53.


$$\begin{array}{l} Q_{1}=0.5(\frac{0.820683-0.341707}{1.207565-0.341707})+(1-0.5)(\frac{0.53-0.216667}{0.73-0.216667})\\ \;=0.27659+0.305195=0.581785 \end{array}$$

If O, S, and D are all considered as cost indices, the risk priority index *Q*_1_ for error 1 is calculated using VIKOR as follows:

$$Q_{1}=v(\frac{S_{1}-S*}{S^{-}-S*})+(1-v)(\frac{R_{1}-R*}{R^{-}-R*}).$$

The calculated values of S*, *S*^−^, R*, *R*^−^, *S*_1_, and *R*_1_ were 0.2907, 1.7133, 0.2167, 0.73, 0.2907, 0.2578, respectively, for VIKOR.


$$\begin{array}{l} Q_{1}=0.5(\frac{0.2907-0.2907}{1.7133-0.2907})+(1-0.5)(\frac{0.2578-0.2167}{0.73-0.2167})\\ \;=0.0401 \end{array}$$

In both situations, the value of *Q*_1_ (*Q*_1_ = 0.581785 or 0.0401) differs from that (*Q*_1_ = 0.675342 [7]) obtained in Mandal’s study. This may be due to calculation errors in the previous study [7]. In the present study, O, S, and D were all considered as cost indices [50], and the other results of Q_*i*_ were calculated and are presented in column 6 of Table 10.

A third comparison was conducted between the proposed method and VIKOR. As shown in Fig 5, the risk priorities for human error obtained using the proposed method and VIKOR were different. Although the prioritization results are not completely consistent, error 11 always had a higher risk priority than the others in this case, indicating the effectiveness and robustness of the proposed method. From Fig 5 and Table 10, the prospect value interval and priority order range obtained via the proposed method were [–2.0148, 0.5196] and 1–19, respectively, which were wider than those of the VIKOR method. This indicates that the proposed framework is effective for ranking human error risk prioritization in crane operations. The differences in the prioritization results obtained via the proposed method and VIKOR are primarily attributed to their distinct mechanisms for prioritizing human error risk in overhead crane operations. The VIKOR method uses the risk value Q to rank the risk priorities of human error, whereas the proposed method ranks risk priorities according to overall prospect values, which can reflect the experts’ risk attitude and psychological behavior. VIKOR considers only the subjective weights of the risk factors, whereas the proposed method involves integration weights combined with ICWGT. Therefore, the risk prioritization results obtained using the proposed method are more rational and practical than those obtained using VIKOR.

## 5. Conclusions

### 5.1. Summary

Considering the shortcomings of existing methods, we propose a model for human error risk prioritization in crane operations based on the ICWGT and CPT. To address the uncertainty in risk assessment related to human error, trapezoidal fuzzy numbers are employed to describe the risk information. Subsequently, the entropy weighting method is used to calculate the objective weights of the three risk factors. The ICWGT is applied to combine the subjective and objective weights. Furthermore, a ranking mechanism based on the CPT was established for evaluating human error risks in crane operations. The overall prospect value is adopted to determine the risk ranking. Finally, a case study involving human error risk priority in overhead crane operations was conducted to validate the proposed method. A comparative analysis indicated that the proposed method can reasonably prioritize human error risk in crane operations.

The key accomplishments and contributions of this study are summarized as follows. First, a risk priority model for human errors in crane operations based on the ICWGT and CPT was developed. Second, the extended FMEA model incorporating the CPT considers experts’ risk attitudes and decision psychology, resulting in a relatively objective and rational risk priority ranking. Third, in the ICWGT combination weighting method, reasonable weights are obtained by separately determining the subjective and objective weights of O, S, and D. The proposed model may be applicable to risk assessments in other industries.

### 5.2. Limitations of study

The proposed method, which incorporates the CPT and ICWGT for human error risk prioritization in crane operations, has limitations. (1) In the risk assessment process, the experts’ attitudes toward risk are dynamic. This implies that different experts may have different attitudes toward risk, which can vary over time. However, the proposed model neglects the dynamic nature of expert opinions during the evaluation process by assuming that the viewpoints of the experts remain unchanged. Consequently, further research is needed to explore the dynamic risk attitudes of experts throughout the assessment process. (2) The proposed method does not consider the potential influence of leaders or experts who may have special connections within the organization. These individuals may have a significant impact on the risk attitudes of experts. Future research should consider the role of these influential figures and explore the extent to which their connections affect experts’ attitudes toward risk. (3) Importantly, the results presented in this paper were obtained from a limited set of case data. Further practical studies are needed to confirm the feasibility and effectiveness of the proposed model. Future research can help verify the applicability of the proposed model across different contexts and provide a more comprehensive understanding of its potential benefits.

### 5.3. Proposed further research

Future studies should explore the following avenues. First, the linguistic evaluation information provided by different team members regarding the risk factors must be aggregated by considering the members’ weights. Second, it may be necessary to not only rank the human error risks of crane operations but also determine their risk grades. Third, hesitant fuzzy sets, spherical fuzzy sets, Pythagorean fuzzy sets, and Z-numbers should be explored to capture experts’ evaluation information more reliably and comprehensively.

## References

[1] Zhu LB, Wu X, Chen H. Study on special equipment safety risk assessment and control measures. China Safety Science Journal. 2014; 24:149–155.

[2] AQSIQ. A report on the national safety status of special equipment in 2021. https://baijiahao.baidu.com/s?id=1730546450481711462&wfr=spider&for=pc.

[3] Ruud S, Åge Mikkelsen. Risk-based rules for crane safety systems. Reliability Engineering and System Safety. 2008; 93:1369–1376.

[4] Aneziris ON.; Papazoglou IA, Mud ML, Damenc M, Kuiperd J, Baksteene H, Alef BJ, Bellamyg LJ, Haleh AR, Bloemhoffd AJ, Posth G, Ohi J. Towards risk assessment for crane activities. Safety Science. 2008; 46:872–884.

[5] Fang Y, Cho YK, Chen J. A framework for real-time pro-active safety assistance for mobile crane lifting operations. Automation in Construction. 2016:367–379.

[6] Huang K, Zhang Y, Li XD. Safety evaluation for deformation structural defect of shipbuilding gantry crane based on fuzzy analytical hierarchy process. Hoisting and Conveying Machinery. 2012;12:51–55.

[7] Mandal S, Singh K, Behera RK, Sahu SK, Raj N, Maiti J. Human error identification and risk prioritization in overhead crane operations using hta, sherpa and fuzzy vikor method. Expert Systems with Applications. 2015; 42(20):7195–7206.

[8] Vukelic G, Pastorcic D, Vizentin G, Bozic Z. Failure investigation of a crane gear damage. Engineering Failure Analysis. 2020;115,104613.

[9] Yu JX, Wu SB, Chen HC, Yu Y, Fan HZ, Liu JH. Risk assessment of submarine pipelines using modified fmea approach based on cloud model and extended vikor method. Process Safety and Environmental Protection.2021; 155:555–574.

[10] Gargama H, Chaturvedi SK. Criticality assessment models for failure mode effects and criticality analysis using fuzzy logic. IEEE Trans. Reliab. 2011; 60:102–110.

[11] Zhang ZB, Ding KQ, Liu GS. Research on function monitoring technology of shore container crane based on FMEA. Hoisting and Conveying Machinery. 2019; 10:62–66.

[12] Sun YT, Qin XR, Zhou ZW, Zhang Q. Failure mode and influence analysis of quay crane heightening construction process based on fuzzy set theory. Chinese Journal of Construction Machinery. 2018;16(1): 1–5.

[13] Fu Y, Qin Y, Wang W, Liu X, Jia L. An extended fmea model based on cumulative prospect theory and type-2 intuitionistic fuzzy vikor for the railway train risk prioritization. Entropy. 2020,22(12):1418. doi: 10.3390/e22121418 33333933 PMC7765447

[14] Li XY, Liu KF, Shu AQ, Ding KQ. FMEA analysis of metallurgical bridge crane based on fuzzy confidence theory and grey correlation decision. Hoisting and Conveying Machinery. 2015;5:7–12.

[15] Das S, Dhalmahapatra K, Maiti J. Z-number integrated weighted vikor technique for hazard prioritization and its application in virtual prototype based eot crane operations. Applied Soft Computing. 2020;94:1–13.

[16] Zhao Z, Li A, Ma R, et al. An improved FMEA method of quay crane heightening based on entropy and fuzzy GRA. Machine Design & Reseach. 2022; 38(5):201–204.

[17] Dang TT, Nguyen NAT, Nguyen VTT, Dang LTH. A Two-Stage Multi-Criteria Supplier Selection Model for Sustainable Automotive Supply Chain under Uncertainty. Axioms 2022; 11:228.

[18] Kumari R, Mishra AR. Multi-Criteria COPRAS Method Based on Parametric Measures for Intuitionistic Fuzzy Sets: Application of Green Supplier Selection. Iran. J. Sci. Technol. Trans. Electr. Eng. 2020; 44:1645–1662.

[19] Zhang J, Yang D, Li Q, Lev B, Ma Y. Research on Sustainable Supplier Selection Based on the Rough DEMATEL and FVIKOR Methods. Sustainability. 2020; 13:88.

[20] Memari A, Dargi A, Akbari Jokar MR, Ahmad R, Abdul Rahim AR. Sustainable Supplier Selection: A Multi-Criteria Intuitionistic Fuzzy TOPSIS Method. J. Manuf. Syst. 2019; 50:9–24.

[21] Menekse A, Camgoz Akdag H. Distance education tool selection using novel spherical fuzzy AHP EDAS. Soft Comput. 2022; 26:1617–1635. doi: 10.1007/s00500-022-06763-z 35095335 PMC8785709

[22] Wang CN, Yang FC, Vo TMN, Nguyen VTT, Singh M. Enhancing Efficiency and Cost-Effectiveness: A Groundbreaking Bi-Algorithm MCDM Approach. Appl. Sci. 2023; 13: 9105.

[23] Wang CN, Dang TT, Nguyen NAT. Location Optimization of Wind Plants Using DEA and Fuzzy Multi-Criteria Decision Making: A Case Study in Vietnam. IEEE Access. 2021; 9:116265–116285.

[24] Li QY, Chen GM. Effectiveness evaluation of missile electromagnetic launch system based on adc model improved by EWM-FAHP-ICWGT. Mathematical Problems in Engineering. 2020;1–19.

[25] Li AH. ICWGT-IVFS multi-level safety assessment model for quayside container crane metal structure. China Safety Science Journal. 2018; 18(12): 129–135.

[26] Wang WZ, Liu XW, Qin Y, Fu Y. A risk evaluation and prioritization method for FMEA with prospect theory and Choquet integral. Saf. Sci. 2018; 110: 152–163.

[27] Tversky A, Kahneman D. Advances in prospect theory:cumulative representation of uncertainty. Journal of risk and uncertainty. 1992; 5:297–323.

[28] Wu YN, Xu CB, Zhang T. Evaluation of renewable power sources using a fuzzy MCDM based on cumulative prospect theory: A case in China. Energy. 2018; 147:1227–1239.

[29] Cheng MY, Tsai HC, Chuang KH. Supporting international entry decisions for construction firms using fuzzy preference relations and cumulative prospect theory. Expert Systems with Applications. 2011;38(12):15151–15158.

[30] Li AH, Zhao ZY. Crane Safety Assessment Method Based on Entropy and Cumulative Prospect Theory. Entropy. 2017;19(1):1–16.

[31] Li XH, Wang FQ, Chen XH. Trapezoidal intuitionistic fuzzy multi-attribute decision making method based on cumulative prospect theory and dempster-shafer theory. Journal of Applied Mathematics. 2014: 1–18.

[32] Cheng MY, Tsai HC, Chuang KH. Selecting the Optimal Micro-Grid Planning Program Using a Novel Multi-Criteria Decision Making Model Based on Grey Cumulative Prospect Theory. Energies. 2018;11(7):1840; doi: 10.3390/en11071840

[33] Curtis IA. Valuing ecosystem goods and services: a new approach using a surrogate market and the combination of a multiple criteria analysis and a Delphi panel to assign weights to the attributes, Ecological Economics. 2004; 50(3):163–194.

[34] Meng B, Chi G. New Combined Weighting Model Based on Maximizing the Difference in Evaluation Results and Its Application, Mathematical Problems in Engineering.2015:1–9.

[35] Zbikowski K. Using Volume Weighted Support Vector Machines with walk forward testing and feature selection for the purpose of creating stock trading strategy, Expert Systems with Applications.2015;42(4):1797–1805.

[36] Chen HY. Combination determining weights method for multiple attribute decision making based on maximizing deviations.System Engineering and Electronics.2004;26(2):194–197.

[37] Zhou YF, Wei FJ. Combination weighting approach in multiple attribute decision making based on relative entropy. Operations Research and Management Science.2006;15(5):48–53.

[38] Mao HB, Zhang FM, Feng H, Zou WG. A method of combination weighting for multiple attribute decision making based on interval estimation. Systems Engineering: Theory and Practice.2007; 27(6):86–92.

[39] Ma J, Fan ZP, Huang LH. A subjective and objective integrated approach to determine attribute weights. European Journal of Operational Research.1999;112(2):397–404.

[40] Fan ZP, Zhang Q, Ma J. An integrated approach to determining weights in multiple attribute decision making. Journal of Management Sciences in china. 1998;1(3): 50–53.

[41] Shi L, Yang SL, Ma Y, Yang Y. A novel method of combination weighting for multiple attribute decision making. Journal of Systems Engineering. 2012;27(8): 481–491.

[42] Lai C G, Chen X H, Chen XY, Wang ZL, Wu XS, Zhao SW. A fuzzy comprehensive evaluation model for flood risk based on the combination weight of game theory. Natural Hazards.2015; 77(2): 1243–1259.

[43] Wang SJ, Fei LJ, Lei YB, Tian W. Two kinds of comprehensive weight combination method applied to irrigation district’s evaluation. Journal of Xi’an University of Technology. 2009; 25(2): 207–211.

[44] Chen NX, Yang QX. Set Pair Analysis of Watershed Water Resources Carrying Capacity Based on Game Theory Combination Weighting. Journal of Irrigation and Drainage. 2013;32(2):81–85.

[45] Xiao SS, Li KM, Ding XH, Liu T. Rock Mass Blastability Classification Using Fuzzy Pattern Recognition and the Combination Weight Method. Mathematical Problems in Engineering. 2015:1–11.

[46] Zhou JG, Wang XW. Game theory and gray incidence degree based appraisement index system for operation effect of regional electricity market. Power System Technology.2007; 31(10):69–73.

[47] Su GN, Fu XQ, Liu TX. Improved Comprehensive Weight Calculated with Game Theory and Its Application in Dam Safety Synthetic Appraisal. China Rural Water and Hydropower. 2014;11:82–85.

[48] Sun LJ, Liu YY, Zhang BY, Shang YW, Yuan HW, Ma Z. An Integrated Decision-Making Model for Transformer Condition Assessment Using Game Theory and Modified Evidence Combination Extended by D Numbers. Energies. 2016;9:1–22.

[49] Liao R, Stanislaw Grzybowski HZ, Yang L, Zhang Y, Liao Y. An integrated decision-making model for condition assessment of power transformers using fuzzy approach and evidential reasoning. IEEE Transactions on Power Delivery. 2011; 26:1111–1118.

[50] Mangeli M, Shahraki A, Saljooghi FH. Improvement of risk assessment in the FMEA using nonlinear model, revised fuzzy TOPSIS, and support vector machine. International Journal of Industrial Ergonomics. 2019;69:209–216.

[51] Young JL, Liu TY, Hwang CL.TOPSIS for MODM. European Journal of Operational Research. 1994; 6: 486–1500.

[52] Wang JQ, Sun T, Chen XH. Multi-criteria fuzzy decision-making method based on prospect theory with incomplete information. Control and Decision. 2009; 24:1198–11202.

[53] Li XW, Wang W, Xu CC, Li Z. Multi-objective optimization of urban bus network using cumulative prospect theory. Journal of Systems Science and Complexity. 2015; 28:661–1678.

[54] Wu Y, Ke Y, Xu C, et al. An integrated decision-making model for sustainable photovoltaic module supplier selection based on combined weight and cumulative prospect theory.Energy. 2019; 181:1235–1251.

